# Long-term clinical outcome of oestrogen receptor-positive operable primary breast cancer in older women: a large series from a single centre

**DOI:** 10.1038/bjc.2011.105

**Published:** 2011-03-29

**Authors:** B M Syed, W Al-Khyatt, S J Johnston, D W M Wong, L Winterbottom, H Kennedy, A R Green, D A L Morgan, I O Ellis, K L Cheung

**Affiliations:** 1 Division of Breast Surgery, University of Nottingham, Nottingham NG5 1PB, UK; 2 Nottingham Breast Institute, Nottingham University Hospitals, Nottingham NG5 1PB, UK; 3 Division of Pathology, University of Nottingham, Nottingham NG5 1PB, UK; 4 Department of Oncology, Nottingham University Hospitals, Nottingham NG5 1PB, UK

**Keywords:** elderly, ER, primary breast cancer, endocrine therapy, surgery

## Abstract

**Introduction:**

A Cochrane review of seven randomised trials (*N*=1571) comparing surgery and primary endocrine therapy (PET) (oestrogen receptor (ER) unselected) shows no difference in overall survival (OS). We report outcome of a large series with ER-positive (ER+) early invasive primary breast cancer.

**Methods:**

Between 1973 and 2009, 1065 older (⩾70 years) women (median age 78 years (70–99)) had either surgery (*N*=449) or PET (*N*=616) as initial treatment.

**Results:**

At 49-month median follow-up (longest 230 months), the 5-year breast cancer-specific survival (BCSS) and OS were 90 and 62%, respectively. Majority (74.2%) died from causes other than breast cancer. The rates (per annum) of local/regional recurrence (<1%) (following surgery), contralateral tumour (<1%) and metastases (<3%) were low. For patients on PET, 97.9% achieved clinical benefit (CB) at 6 months, with median time to progression of 49 months (longest 132 months) and significantly longer BCSS when compared with those who progressed (*P*<0.001). All patients with strongly ER+ (H-score >250) tumours achieved CB and had better BCSS (*P*<0.01). Patients with tumours having an H-score >250 were found to have equivalent BCSS regardless of treatment (surgery or PET; *P*=0.175), whereas for those with H-score ⩽250, surgery produced better outcome (*P*<0.001).

**Conclusion:**

Older women with ER+ breast cancer appear to have excellent long-term outcome regardless of initial treatment. Majority also die from non-breast cancer causes. Although surgery remains the treatment of choice, patients with ER-rich (H-score >250) tumours tend to do equally well when treated by PET. This should be taken into account when therapies are considered.

Advancing age is one of the major risk factors for breast cancer development, with a risk of 1 in 208 at the age of 40 years increasing to 1 in 16 at the age of ⩾70years (Jemal *et al*, 2009), resulting in 33% of breast cancer occurring after 70 years of age (Office for National Statistics, 2005; Cancer Research UK, 2009). The incidence of breast cancer in older women is increasing worldwide and is expected to rise more in the upcoming years, as a result of longer lifespan. There has been minimal representation of older patients in most clinical trials and management-related studies, making the establishment of clear management guidelines difficult. More than 60% of women over the age of 70 years have at least one significant co-morbid condition (angina or myocardial infarct in 25.7%, congestive heart failure in 25.0%, history of stroke in 16.2% and rheumatological conditions in 65.8%, as shown in a study; Fleming *et al*, 1999) that could limit their life expectancy or produce negative impact on their quality of life. The number of co-morbidities has also been shown to be inversely proportional to life expectancy (Fleming *et al*, 1999).

Given the limited life expectancy and senility-associated co-morbidities, surgery may not be appropriate for some older women, or they may not be willing to have surgery because of their associated social problems. Primary endocrine therapy (PET) then becomes a viable option. A recently published audit of breast cancer management showed that 40% of women over 70 years of age were treated with PET in the United Kingdom (Wyld *et al*, 2004). Therefore, it is of prime importance in the current situation to know the efficacy of PET in comparison with surgery and also to identify the group of patients whose treatment outcome from PET may be comparable with that from surgery.

A recent Cochrane review of seven randomised trials (*N*=1571) comparing surgery and PET in women ⩾70 years of age, unselected for oestrogen receptor (ER) status, did not show any difference in the two treatment arms in terms of overall survival (OS) (Hind *et al*, 2007). Older patients are more likely to die from co-morbidities other than breast cancer (Diab *et al*, 2000); hence, OS might not be the actual predictor of disease outcome. The patients with ER-positive tumours respond well to endocrine therapy, which is an important factor and should have been taken into account for such comparison.

Given the background discussed above in terms of biology and co-morbidities impacting on the management approach and outcome, and the lack of high-level large-scale randomised evidence, this study aimed to: (1) describe and analyse the long-term clinical outcome of older women with ER-positive early operable primary breast cancer treated in a single centre regardless of primary treatment; and (2) compare surgery *vs* PET in this cohort.

## Materials and Methods

As part of an ongoing research programme on early operable primary breast cancer in older women, a database was established, including 1709 patients, diagnosed and treated at the Nottingham Breast Unit from 1973 to 2009. The patients were first identified from the archive of paper records (before 1987, *N*=240) and a computerised database (since 1987, *N*=1469) of the Histopathology Department, with clinical information subsequently confirmed in clinical records. All patients were females ⩾70 years of age, who had either ductal carcinoma *in situ* or invasive early operable primary breast cancer ⩽5 cm of clinical size without evidence of distant metastases at the time of diagnosis.

All patients were managed in a dedicated Primary Breast Cancer Clinic for Older Women (the Clinic) following the same clinical guidelines at any time point. For the patients identified in this data set, data were retrospectively collected from case notes, including detailed information of the clinical course of the disease from the date of diagnosis till death or last follow-up. The data set was based on the variables recorded for the primary breast cancer series used to derive the Nottingham Prognostic Index (NPI) (Haybittle *et al*, 1982; Blamey, 1996) for patients treated by surgery. For patients on nonoperative therapy (predominantly PET), clinical responses at 6 months, progression status and time to progression were recorded for all lines of endocrine therapy and best response recorded only for first-line PET.

### Patients

Out of the established data set as described above, all women with ER-positive (defined in the section on tumour characteristics) invasive carcinoma treated by primary surgery (with and without adjuvant endocrine therapy) (*N*=449) or PET (*N*=616) were included in this study. This study has included some patients recruited into two small, previously reported, randomised controlled trials comparing primary tamoxifen *vs* mastectomy as long as they had ER-positive tumours (Willsher *et al*, 1997; Chakrabarti *et al*, 2010). For the analysis of response to PET, only patients with response status at 6 months available (first line *N*=541, second line *N*=209 and third line *N*=62) were included, as per British Breast Group recommendations (Group, 1974).

### Treatment protocol

The treatment protocol had been changing over the period because of evolving clinical evidence and unit policy. Because of the focus of this study, the management protocols given below describe the unit policy only for patients with ER-positive tumours in each period of time.

#### 1973–1999

All patients with a confirmed diagnosis of breast cancer were given the choice of surgery or PET. Patients undergoing surgery had options of mastectomy or breast conservation (wide local excision (WLE)) as appropriate. Axillary surgery was only considered when there were clinically palpable axillary lymph node(s). The patients who underwent WLE were given no further treatment, adjuvant endocrine therapy with or without adjuvant radiotherapy, depending on the clinical judgement of the clinician, based on tumour grade, margin status, degree of ER positivity and nodal status if available.

For patients who opted for PET, tamoxifen was the drug of choice. Patients with contraindications to tamoxifen (e.g., thromboembolic risk) were then offered a nonsteroidal aromatase inhibitor (AI) such as anastrozole, since it became available in 1995. When patients progressed on first-line PET, a number of treatment options were considered, depending on the patient's choice, fitness, previous response to endocrine therapy and development of distant metastases, including surgery, further endocrine therapy or primary radiotherapy.

#### 2000–2009

The treatment protocol was more structured and all cases were discussed at a multidisciplinary team meeting before the patients were seen in the Clinic (which has become a combined surgical/oncology facility during this period).

All patients with a confirmed diagnosis of breast cancer were recommended to have surgery (mastectomy or WLE as appropriate). Axillary surgery (four-node sampling with introduction of blue-dye sentinel node biopsy recently; or level III axillary clearance for node-positive cases proven by ultrasound-guided core biopsy preoperatively) became part of standard surgery. Intact breast irradiation was normally given following WLE unless the patient was on adjuvant endocrine therapy. Post-mastectomy chest wall irradiation was normally given if the tumour had two out of the following three features: grade 3, positive nodal status and vascular invasion. Patients having tumours with an NPI of ⩾3.4 (moderate and poor prognostic groups) received adjuvant endocrine therapy (using tamoxifen that has subsequently been replaced by a nonsteroidal AI). Those having tumours with an NPI between 3 and 3.4 (good prognostic group) were given the option of no adjuvant endocrine therapy or tamoxifen. Those belonging to the excellent prognostic group (NPI <3) did not receive any systemic therapy.

The patients who refused or were deemed unfit for surgery were then given PET as described above.

#### Assessment of co-morbidities

There was no formal tool for co-morbidity assessment used in the Clinic. However, all patients were assessed by a single surgeon with an interest in the management of older women with primary breast cancer. During the most recent decade, the Clinic has evolved into a combined surgical/oncology facility, supported by two breast cancer nurses in order to further improve such assessment. A pilot study to evaluate a cancer-specific comprehensive geriatric assessment (CGA) tool has also commenced recently.

### Follow-up

All patients were followed up in the Clinic. Patients treated surgically were followed-up on a yearly basis or anytime they required consultancy for any new finding. Patients on PET were followed-up at 6-week time after commencement of therapy, and then 3- and 6-month intervals thereafter or anytime as required. Response to PET was assessed clinically by callipers using International Union Against Cancer (UICC) criteria (Hayward *et al*, 1977), with adherence to the British Breast Group recommendations (Group, 1974).

### Tumour characteristics

Pathological information was based on needle core biopsies for all patients, as reported by a single dedicated team of pathologists following the same guidelines at any time point. Positive ER status was defined in majority of cases by histochemical score (H-score) ⩾50, using immunohistochemistry as previously described (Nicholson *et al*, 1986). In the earlier period, charcoal-coated method was used for ER assessment in a few cases (*N*=3) and positivity was defined as 5 fmol of cytosolic protein equivalent to Allred score of 5 (Shousha, 2008).

The grade of the tumour and the axillary stage were recorded from histopathology reports of surgical specimens: grade defined as the cumulative score of nuclear pleomorphism, tubule formation and mitotic count by the modified Scarf-Bloom-Richardson (SBR) criteria (Elston and Ellis, 1991) and categorised as grade 1–3, and stage defined by the quantitative score of positive axillary lymph nodes (LNs): stage 1=0 LN, stage 2=1–3 LNs and stage 3=⩾4 LNs positive.

### Outcome variables

Survival, metastases and contralateral tumour development were recorded for all patients. Breast cancer-specific survival (BCSS) was defined as the time from the date of diagnosis till death from breast cancer. Overall survival was defined as the time from the date of diagnosis till death regardless of cause. The causes of death were categorised into breast cancer deaths, defined as breast cancer as the first cause of death on the certificate, and non-breast cancer deaths, defined as the registered non-breast cancer cause or if the women with unknown causes of death died within 1 year of their last follow-up without clinical evidence of metastases. The causes of deaths were recorded from the case notes and supplemented by information from the general practitioners (GP) for those patients who were discharged back to GP's care because of their preference. This included patients who subsequently died outside Nottingham following their discharge. A total of 41 (3.8%) patients were completely lost from follow-up and were not included in the analysis of survival.

Metastases were defined as the appearance of tumour at a distant site (including contralateral axillary LN, ipsilateral and contralateral supraclavicular LN, bones, liver, lung and so on) and time to metastases was calculated from the date of diagnosis of the primary tumour till the appearance of metastases.

Contralateral tumour referred to the appearance of a new primary in the other breast, and time to contralateral tumour was calculated in the same way as described above.

Patients treated by surgery had additional outcome variables recorded. Local recurrence was defined as the appearance of tumour in the ipsilateral breast after WLE or in the mastectomy flap. Regional recurrence was defined as the appearance of tumour in the ipsilateral axilla. Time to local or regional recurrence was calculated from the time of surgery.

For patients on PET, additional outcome variables were related to the response to therapy, assessed by the UICC criteria (Hayward *et al*, 1977), and time to progression was defined as the time from the commencement of the endocrine agent till the tumour progressed. Clinical benefit (CB) was defined as the achievement of complete response (CR), partial response (PR) or stable disease (SD) for at least 6 months from the commencement of therapy and objective response (OR) was defined as the achievement of CR or PR for at least 6 months from the commencement of therapy.

### Statistical methods

The statistical Package for Social Sciences (SPSS version 16.0, SPSS Inc., Chicago, IL, USA) was used as the data analysis tool. Statistical significance was defined by *P*-value <0.05. Kaplan–Meier was used to analyse survival and other time-dependant variables, log-rank and Wilcoxon tests were used as appropriate. Standard error (s.e.) and 95% confidence interval (CIs) in terms of time were based on Kaplan–Meier. Given the long timespan of the study time, dependant variables were compared at years 5 and 10, using Kaplan–Meier and life table.

## Results

### Patient and tumour characteristics

A total of 1065 women with ER-positive tumours had been diagnosed and treated with early operable primary breast cancer with a median follow-up of 49 months (endocrine therapy 41 months (longest 202 months), surgery 57 months (longest 230 months)). The median age of the study population was 78 years (range 70–99). Women who received PET were relatively older with a median age of 81 years (range 70–99) when compared with 75 years (range 70–90) for patients who had surgery. The majority of women had strongly ER-positive tumours, with an H-score >200 in 54.7% (Table 1). Increasing age was associated with strongly ER-positive tumours (*P*-value <0.01). The median tumour size was 2.8 cm (range 0–5 cm) and most women had larger tumours, >2.0 cm (65.9%) of clinical size (Table 1). Patients in the PET group had larger tumours with a median size of 3.0 cm (<2 cm=25%, >2 cm=75%), whereas in the surgery group the median size was 2.3 cm (<2 cm=46%, >2 cm=56% *P*-value <0.001). The grade, histological types and axillary stage of the disease reported on surgical specimens are summarised in Table 1. Most patients had grade 2 tumours (51.5%) and stage 1 (61.2%) disease. Ductal carcinoma of no special type (NST) was the most common histological type (59.1%), followed by tubular mixed and lobular types.

### Treatment pattern

Over these three decades, a larger proportion of women received PET (57.8% (*N*=616)) when compared with surgery (42.2% (*N*=449)). Patients who had primary endocrine therapy tended to be older and had larger tumour size, whereas the distribution of ER positivity was similar (Table 1). Specifically during the period of 1973–1999, 301 and 128 patients had PET and surgery, respectively. Recently (2000–2009), 315 and 321 patients were treated by PET and surgery, respectively. The details of surgery and adjuvant therapy are summarised in Table 1. The majority of patients had adjuvant endocrine therapy (75.9% (*N*=341)).

Tamoxifen was the most commonly used first-line agent for patients on PET (69.3% (*N*=427)), followed by anastrozole.

### Outcome

#### Whole series

*Survival*: The 5-year OS and BCSS were 62 and 90%, respectively, regardless of the treatment given. Most of the women in the cohort died from non-breast cancer causes (74.2%); this proportion further increased to 82.9% in the age group ⩾80 years. The data for causes of death are presented in Table 2.

#### Surgery group

*Local and regional recurrence*: The overall rates of local and regional recurrences were low, at 4.7 and 3.1%, respectively (Table 3). There was no significant difference in the rate of and time to local recurrence following mastectomy and WLE (log-rank *P*-value=0.709) and there was no significant difference of the rate of and the time to regional recurrence in the patients undergoing axillary surgery when compared with those who did not have any axillary surgery (log-rank *P*-value=0.732).

#### PET group

*First line*: A total of 616 women were put on PET; 541 patients had their response at 6 months available for analysis and 5 women (0.9%) did not tolerate the therapy (two were on anastrozole and three on tamoxifen). The overall CB was 97.9% with 2.1% (*N*=11) progression within 6 months. The response at 6 months is described in Table 4. Eventually, 519 women achieved CR, PR or SD (CR (*N*=161 (31%)), PR (*N*=182 (35.1%)) or SD (*N*=176 (33.9%)) and the overall eventual progression rate was 45.0% (*N*=244). The median time to progression was 49 months (range 4–132 months). The median duration of CB was 30 months with the longest duration of 180 months (range 7–180 months). A total of 275 patients needed to change their first line of therapy because of eventual progression of disease or intolerance, 90.4% (*N*=254) were switched to second line of endocrine therapy, whereas 7.1% (*N*=20) had surgery and 2.5% (*N*=7) had primary radiotherapy.

*Second line*: Out of the 254 patients on second-line PET, the response at 6 months was available for 209 patients (Table 4). Most of the tumours remained stable (55.0%) and the overall CB was 95.7% with an eventual progression rate of 51.9%. The median time to progression was 30 months (range 3–144 months) and the median duration of CB was 20 months (range 7–144) months). A total of 117 women needed third-line therapy, 74.4% (*N*=87) were switched to third-line endocrine therapy, 14.5% (*N*=17) had surgery, 9.4% (*N*=11) had primary radiotherapy whereas 1.7% (*N*=2) had a combination of endocrine therapy and radiotherapy.

*Third line*: A total of 87 women were put on third-line PET and the response at 6 months was available in 62 women (Table 4). The CB rate at 6 months was 87.1% (*N*=54) with a median time to progression of 20 months (range 1–52 months) and a median duration of CB of 16 months (range 7–85 months).

#### Association of response to PET with H-score

**Figure 1:**
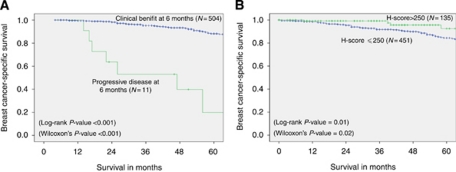
Breast cancer-specific survival of early operable primary breast cancer in older women (⩾70 years of age) – surgery *vs* primary endocrine therapy. (**A**) Clinical benefit (CB) *vs* progressive disease (PD) at 6 months. (**B**) H-score⩽250 *vs* >250.

The women who achieved CB at 6 months had statistically significantly better BCSS when compared with those who progressed, although the number of the latter was small (Figure 1A). Patients with strongly ER-positive tumours (H-score >250) responded well to PET and all of them achieved CB at 6 months (Table 4). The same patients had significantly better BCSS, with the rate of 93% at 10-year when compared with 71% if the H-score was ⩽250 (log-rank *P*-value=0.012; Figure 1B).

### Surgery *vs* PET

#### Contralateral tumours and metastases

The overall rate of contralateral tumour development was 2.1% (surgery=3.4% and PET=1.2%, *P*-value <0.01) and the overall rate of metastases was 10.5% (surgery=9.2% and PET=11.4%, *P*-value=0.143). The data for contralateral tumour and metastases development are summarised in Table 3.

#### BCSS

**Figure 2:**
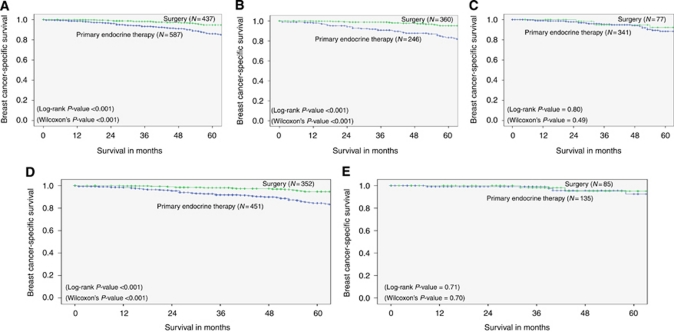
Breast cancer-specific survival of early operable primary breast cancer in older women (⩾70 years of age) treated with primary endocrine therapy. Surgery *vs* primary endocrine therapy – (**A**) whole series; (**B**) age group 70–79 years; (**C**) age group ⩾80 years; (**D**) H-score<250; and (**E**) H-score⩾250.

The 5-year BCSS was 84 and 95% in the PET and surgery groups, respectively (log-rank *P*-value <0.001; Figure 2). When comparing with those receiving PET, the patients having surgery had better BCSS in the age group <80 years (5-year BCSS=83 *vs* 95% in PET *vs* surgery groups respectively, log-rank *P*-value <0.001), whereas in women ⩾80 years there was no significant difference observed between the treatment arms (5-year BCSS=88 *vs* 92% in PET *vs* surgery groups, log-rank *P*-value=0.800; Figure 2). Similar results were observed when only patients who had adjuvant endocrine therapy following surgery were compared with those having PET.

It was found that the patients who had strongly ER-positive tumours (H-score >250) had the same BCSS regardless of treatment (5-year BCSS=93 *vs* 95% in PET *vs* surgery groups, log-rank *P*-value=0.715), whereas if H-score was ⩽250, surgery appeared to produce better outcome (5-year BCSS=84 *vs* 95% in PET *vs* surgery groups, log-rank *P*<0.001; Figure 2).

## Discussion

The results of the study showed a preponderance of relatively lower-grade and ER-rich tumours in older women. Across a period of more than three decades, mastectomy was the most common surgical procedure done and adjuvant endocrine therapy was given in the majority of cases regardless of the type of surgery. The rates of local and regional recurrence, contralateral tumour development and metastases were low. The overall response to PET appeared excellent with >97% CB within 6 months. All women with tumours having an H-score >250 responded well to PET and achieved CB at 6 months. The outcome of surgery and PET in terms of BCSS appeared similar in women >80 years of age or in those with tumours having an H-score >250.

Surgery has been the standard management for early operable primary breast cancer regardless of age and tumour biology. The studies comparing surgery and PET in older women showed better local control of the disease when they were surgically treated, but none of them reported any uncontrollable local disease in the PET arm. Furthermore, as shown in the meta-analysis of seven randomised trials, there was no significant difference in the OS in two treatment arms (Hind *et al*, 2007). Although measuring the OS in older women has an advantage of assessing the influence of co-morbidities, BCSS on the other hand helps to measure tumour behaviour as it takes deaths due to breast cancer specifically into account. Among the seven randomised trials mentioned, with the exception of the GRETA trial, all compared both treatment arms in terms of OS. The GRETA trial, after a median follow-up of 80 months, did not show any difference in BCSS between surgery and PET (Mustacchi *et al*, 2003).

The randomised controlled trials included in the above-mentioned Cochrane review were unselected for ER status, with the exception of the Nottingham II trial, which included patients with ER-positive tumours (H-score ⩾100; Willsher *et al*, 1997). Tamoxifen was the agent used in all these trials to compare with surgery; however, emerging evidence on AIs showed similar or better response when compared with tamoxifen. These drugs have the potential to produce improved outcome, including local control, with PET. The outcomes of this cohort study are consistent with those reported in the Nottingham II trial (selected for ER with H-score ⩾100 instead of ⩾50 as in this present larger study) in terms of CB. The CB rates at 6 months were 97 and 97.9%, respectively in the Nottingham II trial and the present study (Willsher *et al*, 1997).

Although available literature suggests surgery as the standard treatment, this approach remains questionable in older patients with limited life expectancy, significant number of co-morbidities and social dependency. Recently, in the United Kingdom, a nationwide audit of the pattern of management of breast cancer showed that 40% of older patients received PET (Wyld *et al*, 2004). The concerns in the management of older patients with breast cancer are not only the control of the disease, but also the quality of life; hence, it is crucial to understand the criteria to select patients who are likely to benefit from PET rather than surgery. The Endocrine ± Surgical Therapy for Elderly women with Mammary cancer (ESTEeM) trial was designed to answer these questions. The trial was established in the United Kingdom aiming to randomise women >75 years of age with ER-positive early operable primary breast cancer to have either surgery followed by adjuvant anastrozole or primary anastrozole as initial treatment. Unfortunately, it was prematurely closed because of slow recruitment.

Given the challenges in recruiting older patients into randomised controlled trials, which have been the gold standard leading to a change in practice, non-randomised, observational or even long-term retrospective studies might be options to be considered as the best possible evidence to set clinical guidelines for this group of patients.

This study, a retrospective review with long-term follow-up and large sample size, from a single centre, attempted to address this issue. Comparatively older patients opt to have PET because of co-morbidities, which prevent them from having surgery. The study by Tang *et al* (2011) showed that patients on PET were on average 7 years older than those patients who had surgery; the related factors were the number of co-morbidities and their own choice. This might explain the slightly larger tumour size seen in the group of patients treated by PET, although the distribution appears similar in both groups in terms of the degree of ER positivity (Table 1).

As age advances, patients are less likely to die from their cancer, with decreasing breast cancer mortality. The study by Diab *et al* (2000) showed 29% of breast cancer deaths in women over 85 years of age and Chapman *et al* (2008) showed 28% of breast cancer deaths in the MA.17 trial. Our study showed similar findings in that majority of deaths were because of non-breast cancer causes and this became even more pronounced for women >80 years.

The rates of local and regional recurrences were low in our study population, not influenced by the type of surgery. The Nottingham II trial showed 4% of local recurrence rate at a median follow-up of 3 years and 11% of regional recurrence in the women with ER-positive tumours without axillary surgery. This might be because of the influence of adjuvant endocrine therapy. The randomised controlled trials comparing axillary surgery and no axillary surgery in the elderly women with early operable primary breast cancer have also shown no significant difference in outcome (Martelli *et al*, 2005; Group IBCS, 2006).

Despite the fact that over half of our patients who had breast-conserving surgery did not receive postoperative radiotherapy, their local recurrence rates remained low, especially when they were taking tamoxifen together as previously reported (Valassiadou *et al*, 2007). This observation is also supported by a randomised controlled trial (Hughes *et al*, 2004, 2010).

Advancing age seems to be associated with strongly ER-positive tumours; Cheung *et al* (2008) showed that 44.4% of tumours had H-score >200 (the peak H-score being 200–300) and 70.3% had >80% of cells stained positive for ER in the patients >70 years of age. This was in contrast to the peak H-score of 100–200 among ER-positive tumours in women <70 years.

Although ∼10% of patients were not available for follow-up assessment at 6 months after having been started on PET, this remains a large series (*N*=541), when compared with the literature – even the Cochrane review of seven randomised trial includes 1571 patients treated by either surgery or PET (Hind *et al*, 2007). Our study therefore provides an important contribution to the literature in this area.

The response to PET appeared excellent for each line of therapy with minimal rate of intolerance to therapy and considerably long time to progression and duration of CB. The CB rate of PET in the advanced breast cancer setting was shown to be ∼70%, (Robertson *et al*, 1997) but in this study, which included very rich ER early operable cancer, the response appeared superior (>97%). The response to PET appeared to be associated with the degree of ER positivity and all those with strongly ER positive tumours (H-score >250) achieved CB at 6 months and had better BCSS when compared with those who progressed at 6 months with H-score ⩽250. In this population with ER-rich tumours, PET was found to produce comparable outcome as surgery in terms of BCSS. The achievement of CB at 6 months has been demonstrated as an indication to continue PET and patients were found to have better survival than those who progressed early (Robertson *et al*, 1997; Willsher *et al*, 1997). This finding has a potential value in guiding the use of neoadjuvant endocrine therapy.

Older women with ER-positive invasive early operable primary breast cancer appear to have excellent long-term clinical outcome regardless of initial treatment. Although surgery remains the primary treatment of choice, patients with ER-rich tumours (H-score >250, also more commonly found with increasing age) tend to do equally well when treated by PET. This study has focussed on the analysis of long-term outcome in terms of BCSS, which is a reflection of tumour behaviour. Within this context, we have already demonstrated comparable outcome between surgery and PET for patients with ER-rich tumours. Comparison of OS is deemed inappropriate in this study given the nonrandomised nature. However, given that most patients die from non-breast cancer causes, affecting the OS, it would not be difficult to appreciate the potential value of PET, especially in patients with ER-rich tumours, as well as a number of significant co-morbidities further shortening their life expectancy (the median time of progression on PET regardless of H-score in this study was 49 months).

With the growing elderly population, given the complexity of their problems and the differences in biology of breast cancer, older women with newly diagnosed primary breast cancer deserve a more detailed work up in order to optimise their management.

## Figures and Tables

**Table 1:** Patient and tumour characteristics and treatment pattern in older women with early operable primary breast cancer

	**Number of patients**	**Percentage (%)**	**PET, *N* (%)**	**Surgery, *N* (%)**
*Age (years)*	*N*=1065		*N*=616	*N*=449
70–79	624	58.6	253 (41.1)	371 (82.6)
⩾80	441	41.4	363 (58.9)	78 (17.4)
				
*Clinical size of tumour (cm)*	*N*=1059		*N*=612	*N*=447
0 (Screen detected)	78	7.4	9 (1.5)	69 (15.4)
0.1–2	283	26.7	147 (24.0)	136 (30.4)
2.1–5	698	65.9	456 (74.5)	242 (54.1)
				
*H-score*	*N*=1062		*N*=614	*N*=448
50–100	46	4.3	27 (4.4)	19 (4.2)
101–200	435	41.0	241 (39.3)	194 (43.3)
201–300	581	54.7	346 (56.4)	235 (52.5)
				
*Histological characteristics and treatment pattern of surgery group*				
*Histological grade*	*N*=431			
1	84	19.5		
2	222	51.5		
3	125	29.0		
				
*Axillary stage*	*N*=353			
1	216	61.2		
2	110	31.2		
3	27	7.6		
				
*Histological types*	*N*=445			
Ductal carcinoma (NST)	263	59.1		
Tubular mixed	63	14.2		
Lobular carcinoma	61	13.7		
Mucinous carcinoma	21	4.7		
Other types	37	8.3		
				
*Types of surgery*	*N*=449			
Mastectomy	255	56.8		
WLE	194	43.2		
				
*Adjuvant therapy with mastectomy*	*N*=255			
AdjET only	157	61.6		
AdjRT only	3	1.2		
AdjET+AdjRT	42	16.5		
No adjuvant therapy	53	20.8		
				
*Adjuvant therapy with WLE*	*N*=194			
AdjET only	97	50.0		
AdjRT only	35	18.0		
AdjET+AdjRT	45	23.2		
No adjuvant therapy	17	8.8		

Abbreviations: AdjET=adjuvant endocrine therapy; AdjRT=adjuvant radiotherapy; NST=no special type; PET=primary endocrine therapy; WLE=wide local excision.

**Table 2:**
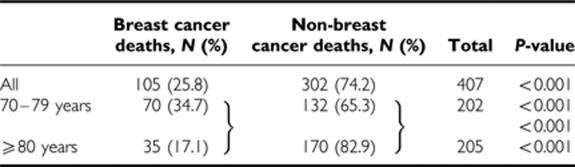
Causes of death by age groups in older women (⩾70 years of age) with early operable primary breast cancer

**Table 3:** Outcome of oestrogen receptor-positive early operable primary breast cancer in older women (⩾70 years of age)

**Event**	**Overall rate (%)**	**Average rate per annum (%)**
*Surgery group only*		
*Local recurrence*		
Whole surgery group	4.7	1.0
Mastectomy	5.1	0.9
WLE	4.2	1.4
*Regional recurrence*		
Whole surgery group	3.1	0.5
Axillary surgery group	2.4	0.5
No axillary surgery group	4.7	0.5
		
*All patients*		
*Contralateral tumour development*		
Whole group	2.1	0.7
Surgery±AdjET	3.4	1.0
Surgery+AdjET	2.7	1.0
PET	1.2	0.4
*Metastases*		
Whole group	10.5	2.0
Surgery±AdjET	9.2	1.9
Surgery+AdjET	9.1	2.0
PET	11.4	2.2

Abbreviations: AdjET=adjuvant endocrine therapy; PET=primary endocrine therapy; WLE=wide local excision.

**Table 4:** Outcome of primary endocrine therapy (response at 6 months) in older women with oestrogen receptor-positive early operable primary breast cancer

	**Response at 6 months**				
**Line of endocrine therapy**	**CR, *N* (%)**	**PR, *N* (%)**	**SD, *N* (%)**	**PD, *N* (%)**	**CB, *N* (%)**
First line (*N*=541)	66 (12.3)	189 (35.5)	270 (50.4)	11 (2.1)	525 (97.9)
Second line (*N*=209)	46 (22.0)	39 (18.7)	115 (55.0)	9 (3.8)	200 (95.7)
Third line (*N*=62)	14 (22.6)	4 (6.5)	36 (58.1)	8 (12.9)	54 (87.1)
					
	**Response at 6 months**				
**H-score**	CB,* *N* (%)			PD,* *N* (%)	
⩽250	403 (97.3)			11 (2.7)	
>250	121 (100)			0 (0)	

Abbreviations: CB=clinical benefit; CR=complete response; PD=progressive disease; PR=partial response; SD=stable disease.

^*^P-value=0.058.
